# Traumatic Transection of Pancreas at the Neck: Feasibility of Parenchymal Preserving Strategy

**DOI:** 10.4021/gr2010.02.163w

**Published:** 2010-03-20

**Authors:** Rudra Prasad Doley, Thakur Deen Yadav, Mandeep Kang, Ashwani Dalal, Mayank Jayant, Rajeev Sharma, Jai Dev Wig

**Affiliations:** a Department of General Surgery, Postgraduate Institute of Medical Education and Research, Chandigarh, India; b Department of Radiodiagnosis, Postgraduate Institute of Medical Education and Research, Chandigarh, India; c Government Medical College, Chandigarh, India

**Keywords:** Pancreas, Pancreatic injuries, Abdominal injuries, Organ preservation, Pancreatic anastomosis

## Abstract

**Background:**

To assess the feasibility and safety of a pancreas preserving operative technique in the management of isolated complete pancreatic neck transection following blunt abdominal trauma.

**Methods:**

Two patients with isolated blunt fracture of the pancreatic neck underwent pancreas preserving procedure comprising of oversewing of the proximal pancreas and Roux-en-Y pancreatico jejunostomy to the distal remnant. A feeding jejunostomy tube was placed for postoperative nutritional support in these patients. Both patients received subcutaneous octreotide 300 µg/day.

**Results:**

Their ages ranged from 15 years to 20 years, mode of injury was bicycle handle-bar injury (n = 2). Both had pancreatic transection at neck in the line of superior mesenteric vessels. One had ascites. These patients had pancreas parenchyma preserving surgery – internal drainage of the left remnant in a Roux-en-Y jejunal loop. The postoperative course was uneventful in these and both are well on follow-up.

**Conclusions:**

Pancreas preserving strategy – suture of head side of pancreas and an internal drainage of left remnant with a Roux-en-Y jejunal loop is feasible and safe and should be considered in selected cases. Substantial amount of normal pancreatic parenchyma is preserved.

## Introduction

Complete transection of the pancreas at pancreatic neck following blunt trauma is uncommon [1-3]. Increased morbidity and mortality result from associated vascular injuries and main pancreatic duct disruption [4, 5].

Management strategies described are distal pancreatectomy with splenectomy [2, 6], spleen preserving distal pancreatectomy, primary repair of the pancreas and main pancreatic duct [7], conservative approach [8], endoscopic stenting [9, 10], and pancreas parenchyma preserving surgical approach [11-13].

Distal pancreatectomy and splenectomy have been the standard of care for patients with blunt pancreatic transection. Distal pancreatectomy to the left of superior mesenteric vessels is associated with loss of significant amount of normal pancreatic parenchyma, thereby increasing the risk of pancreatic insufficiency [14, 15]. Splenectomy is associated with a lifelong risk of infectious and hematologic morbidity [16, 17]. Endocrine and exocrine pancreatic insufficiency, and infectious and hematological complications following distal pancreatectomy and splenectomy have prompted the pancreas and spleen preserving strategies for the management of this uncommon injury [16]. This retrospective study evaluates the feasibility and safety of pancreas parenchyma preserving option for blunt isolated pancreatic neck transection.

## Patients and Methods

**Figure 1:**
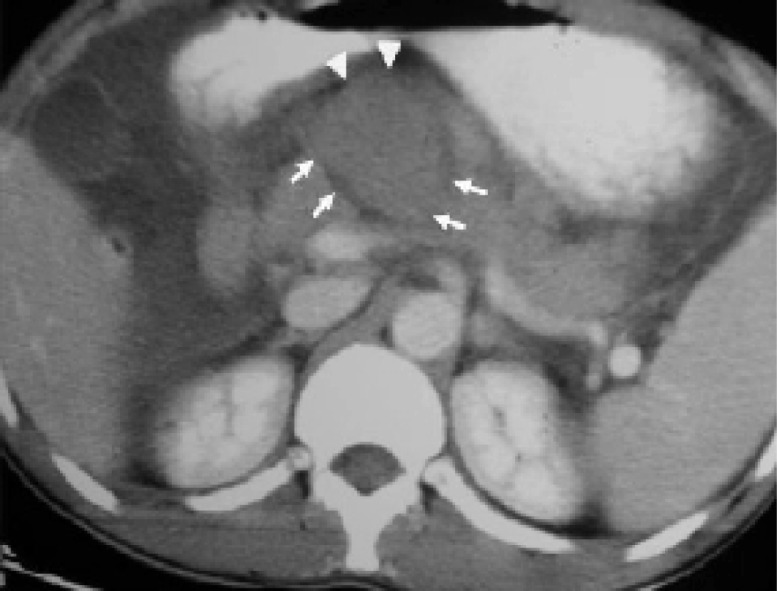
CECT abdomen axial image reveals complete transection between head and neck of pancreas with a rounded hypoechoic mass suggestive of hematoma separating the two fractured fragments (arrows). Note the fluid surrounding the hematoma (arrow heads).

This is a retrospective review of two patients with isolated pancreatic neck transection managed between July 2005 to March 2009. These patients underwent emergency pancreas preserving surgery for isolated pancreatic neck fracture in the line of superior mesenteric vessels. Contrast enhanced computed tomography (CT) in these patients demonstrated the fracture (Fig. 1). An endoscopic retrograde cholangiopancreatography was not attempted in these patients.

### Technique

**Figure 2:**
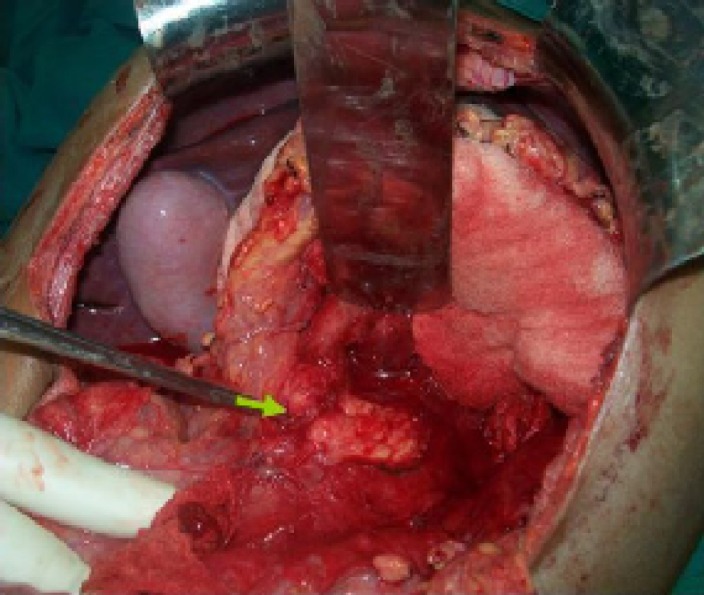
Intraoperative photograph shows the pancreatic fracture (arrow)

**Figure 3:**
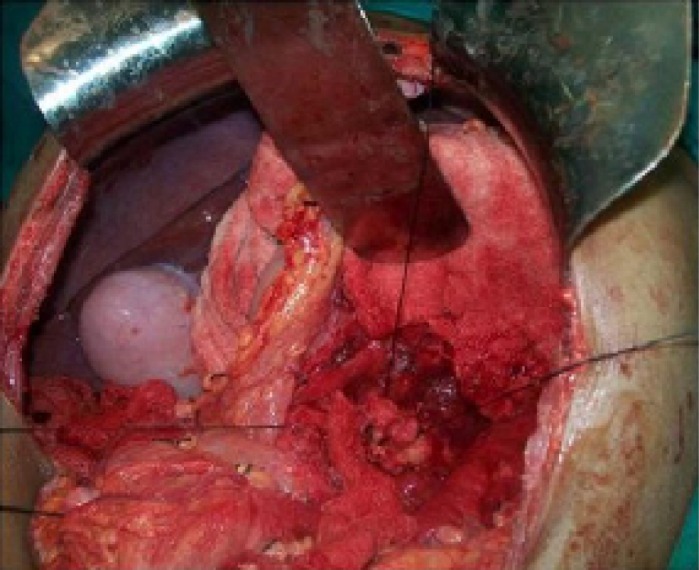
The distal pancreas is lifted for a distance of 2 - 3 cm.

**Figure 4:**
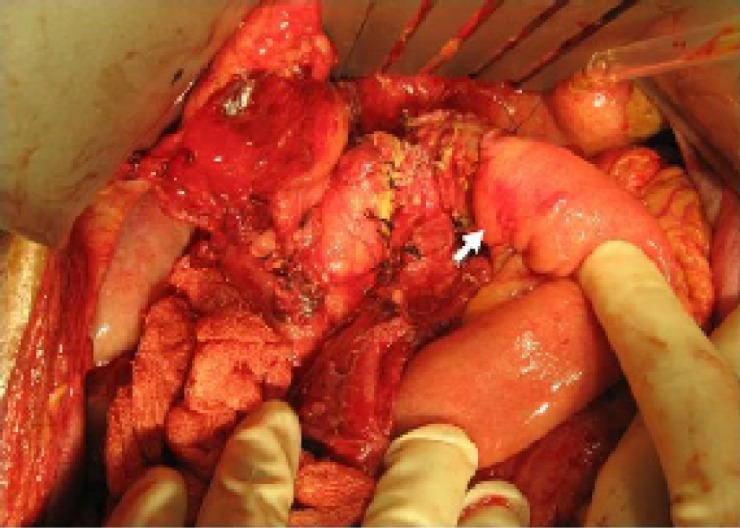
Anastomosis of the distal pancreas to Roux-en-Y jejunal loop.

The lesser sac was approached after ligating and dividing the gastrocolic omentum. The entire pancreas was visualized and assessed. The injury was carefully evaluated (Fig. 2) by ligation and division of remaining pancreatic attachments. The exposed superior mesenteric vein and the portal vein were carefully inspected. The proximal pancreatic head was oversewn with interrupted nonabsorbable sutures after suture ligation of the pancreatic duct. The distal end was mobilized off the superior mesentric vein, splenic vein and portal vein by ligating and dividing small posterior pancreatic vessels which were meticulously isolated. This allowed elevation of distal pancreas by approximately 2 - 3cm for a safe pancreatico-jejunal anastomosis (Fig. 3). Reconstruction of the distal pancreatic remnant was accomplished by Roux-en-Y pancreaticojejunostomy (Fig. 4). An invaginated anastomosis of the distal pancreas into the gut was fashioned using fine, interrupted nonabsorbable sutures.

**Figure 5:**
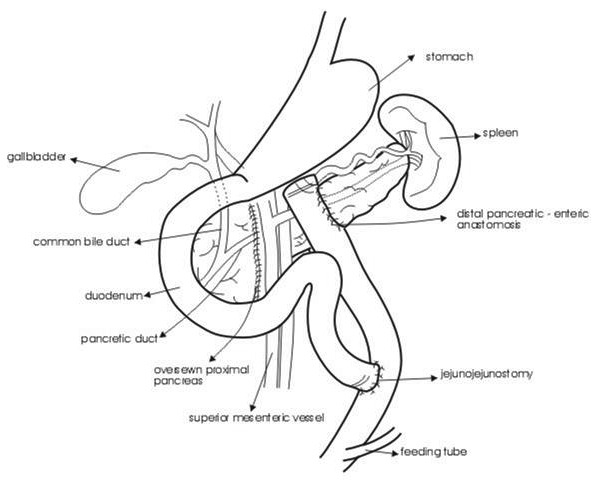
Line diagram describing the operative details.

The operation was completed by an end-to-side jejunostomy distal to the pancreaticojejunostomy (Fig. 5). A 16 Fr portex drain was left in place to adequately control potential leakage from both the proximal stump and the distal pancreatico-jejunostomy.

Postoperative octreotide was administered subcutaneously at a dose of 100 µg 8 hourly. In these patients it was continued for 7 - 10 days in an attempt to decrease the output of pancreatice juice.

## Results

The clinical details of these patients are listed in Table 1. Their ages ranged from 15 years to 20 years, and sustained bicycle handle-bar injury. The clinical presentation was abdominal distention (n = 1), and epigastric tenderness (n = 2). These patients presented within 2 hours to 24 hours. CECT abdomen at admission revealed pancreatic parenchymal transection.

**Table 1:** Patients Demographic Characteristics

Parameter	Case 1	Case 2
Age (years)/Sex	15/Male	20/Male
Duration	2 weeks	24 hours
Mechanism	Bicycle handle-bar	Bicycle handle-bar
Clinical presentation	Ascites, dehydration, Tense abdomen	Epigastric tenderness
CT abdomen	Ascites, Transection neck	Transection neck
Operative findings	Ascites, complete transection	Hematoma complete transection
Operative procedure	Pancreaticojejunostomy	Pancreaticojejunostomy
Outcome	Recovered	Recovered

These patients had conservative surgery – the proximal remnant was oversewn with interrupted nonabsorbable sutures, and a Roux-en-Y pancreatico jejunostomy was performed on the left remnant.

Both these patients survived following pancreas preserving surgery and are well on follow-up ranging from 9 months to over two years.

## Discussion

Complete pancreatic transection is rare and usually occurs in the line of superior mesenteric vessels at the neck of the gland, though it has been reported to occur in the body or tail of the pancreas [6, 18]. In all our patients the injury was confined to the neck of the pancreas. These patients may have associated injury to superior mesenteric portal vein axis. Patients affected by associated vascular injuries are rarely described [5]. Bradley et al [19] reported this association in only 3 of 101 cases. Associated vascular injury carries a high mortaility [20] and most deaths are due to exsanguinating haemorrhage from injury to portal vein, splenic vein, or inferior vena cava [19]. Massive ascites following initial trauma is rarely reported [21]. One of our patients presented with massive ascites two weeks following a bicycle handle-bar injury. Early surgical intervention in patients with pancreatic neck transection and ductal disruption reduces pancreas related morbidity [22]. Transection at this site needs surgical intervention using either resection or internal drainage techniques depending on hemodynamic stability and associated injuries.

Various options are distal pancreatectomy with splenectomy [2, 23-25], distal pancreatectomy with splenic preservation [1], anastomosis of the duct with salvage of the whole organ [26], primary repair of the pancreas and pancreatic duct [7, 27], pancreas preserving Roux-en-Y pancreaticojejunostomy or pancreaticogastrostomy to distal segment [12, 15, 28], and endoscopic transpapillary stent insertion [29, 30]. Exploration and drainage alone result in fistulae, abscesses, pancreatitis, or necrosis as severe complications [18, 31].

The role of pancreatic duct stent is uncertain in patients with complete transection [10, 22, 29], even though there are reports documenting its effectiveness in patients with duct disruption [30]. Pancreatic duct stenting may avert surgical resection when the major pancreatic duct is disrupted but not obstructed [9]. Eventually mild to severe ductal stricture results necessitating intervention [10]. The role of primary repair of the pancreas and pancreatic duct has yet to be defined [7]. Somatostatin analogue therapy may have an important role in management and reduces peripancreatic inflammation and necrosis [24]. It is reported that prophylactic use of octreotide was associated with no pancreatic sequelae [32]. Though there are reports of successful non-operative management in patients with disruption of the main pancreatic duct [33-35], others have reported development of complications resulting in long-term morbidity [22, 35].

Distal pancreatectomy with splenectomy is associated with loss of significant amount of normal pancreatic parenchyma [1, 15] and can lead to long-term pancreatic insufficiency and postsplenectomy infections.

Endocrine impairment after standard left resection is reported in 17% to 85% of patients [36, 37]. The rationale for spleen preserving procedure is preventing postsplenectomy infectious complications. A recent study has shown that following splenectomy, patients had a significantly higher rate of infectious complications (28% versus 9%, p = 0.01), and severe complications (11% versus 2%, p = 0.05) compared with those who had splenic preservation [38]. Other studies have also suggested that spleen preservation should be attempted in patients with benign distal pancreatic disease [16, 39]. Preservation of the spleen may not be always technically feasible as it is more time consuming and associated with increased blood loss [38]. Another study found complication rate twice as high in the splenic preservation group as compared to splenectomy group [40].

In recent years pancreas preserving strategy has been introduced for benign or low grade malignant tumours in the neck or proximal body of the pancreas [36, 41, 42], chronic pancreatitis [43] and for blunt pancreatic neck rupture [1, 12, 44]. Pancreas preserving approach is feasible, safe and appropriate for isolated pancreatic neck transection. Different techniques for reconstruction have been adopted: jejunal anastomosis to both the proximal and distal stump or only to distal stump, or distal pancreaticogastric anastomosis [15, 37, 45]. Reconstruction of the distal pancreatic remnant accomplished with a pancreatico-jejunostomy or pancreatico-gastrostomy preserves as much normal pancreas as possible. The potential risks of endocrine and exocrine insufficiency following removal of more than 50% of normal pancreas are thus prevented [12, 15, 46]. Preserving pancreatic parenchyma depends on hemodynamic stability of the patient and dense inflammatory changes in the region of the neck of the pancreas.

The procedure though technically demanding maintains the function of the pancreas. In recent studies, none of the patients had exocrine or endocrine insufficiency [37, 47, 48]. New onset diabetes is reported in less than 8% [45, 49].

Hirono et al [50] analyzed the frequency of postoperative onset of diabetes mellitus and long-term changes in body weight in patients with a central pancreatectomy and compared with distal pancreatectomy group. The rate of new onset diabetes mellitus was minimal (4.7% versus 35%, p = 0.0129), and the body weight in distal pancreatectomy group was significantly lower than that in central pancreatectomy group at one and two years after surgery (1 year, p < 0.0001, 2 years p = 0.0055). In another study comparing middle pancreatectomy with extended left pancreatectomy, the incidence of new endocrine (4% vs 38%, p = 0.0001) and exocrine insufficiency (5% versus 15.6%, p = 0.039) were significantly higher in extended left pancreatectomy group [36]. Ocuin et al [51] have reported that extended left pancreatectomy group had a higher rate of new onset diabetes mellitus (57% versus 11%, p = 0.04) as compared to central pancreatectomy. New onset exocrine insufficiency was not significantly different between the two groups (p = 0.62).

Both of our patients had no morbidity following parenchyma preserving strategy. There are reports that this strategy is associated with more complications than left pancreatectomy. Ocuin et al [51] reported more complications with central pancreatectomy (92% versus 39%) but there was no significant difference in major complications (38% versus 17%, p = 0.17).

Zhou et al [28] reported a 37.5% morbidity rate and no mortality. The most common complication is a pancreatic leak [52, 53]. There is a possibility of a leak from the closed cut edge of the pancreatic head, or from pancreaticojejunostomy [41, 53]. A normal soft pancreas, and a small main pancreatic duct are risk factors for pancreatic leak. There are conflicting reports regarding the pancreatic leak rate: some reporting leak rates ranging from 6% to 50% [28, 47-49, 53-55], while no leak has been reported in some series [37, 44]. Rotellar et al [41] have reported that the method of pancreatic transection seems to be a decisive factor in the incidence of pancreatic head fistulas. The type of reconstruction – pancreaticogastrostomy versus pancreaticojejunostomy, and the use of a stent did not affect the rate of complications [36]. Most of the pancreatic fistulas may be managed nonoperatively [56]. The reported rates of pancreatic fistula following distal pancreatectomy are 30% to 51% [36, 56]. There is no significant difference in the rate of fistula formation between different stump closure methods following distal pancreatectomy and reduction of pancreatic fistulas after distal pancreatectomy remains an unsolved challenge [57].

In conclusion, the reported surgical strategy is an effective pancreas parenchyma preserving procedure with no postoperative pancreas – related morbidity or mortality. This option may be successfully applied in the emergency setting as a definite surgical procedure and is a promising alternative to distal pancreatectomy and splenectomy for this complex pancreatic injury.
